# Polysaccharide Extracted from* Laminaria japonica* Delays Intrinsic Skin Aging in Mice

**DOI:** 10.1155/2016/5137386

**Published:** 2016-04-06

**Authors:** Longyuan Hu, Jia Tan, Xiaomei Yang, Haitao Tan, Xiaozhen Xu, Manhang You, Wu Qin, Liangzhao Huang, Siqi Li, Manqiu Mo, Huifen Wei, Jing Li, Jiyong Tan

**Affiliations:** ^1^Department of Physiology, Guangxi Medical University, Nanning 530021, China; ^2^Center of Translational Medicine, Guangxi Medical University, Nanning 530021, China; ^3^First Clinical Medical College, Guangzhou Medical University, Guangzhou 511436, China; ^4^Eighth Affiliated Hospital, Guangxi Medical University, Guigang 530007, China

## Abstract

This study aimed to determine the effect of topically applied* Laminaria* polysaccharide (LP) on skin aging. We applied ointment containing LP (10, 25, and 50 *μ*g/g) or vitamin E (10 *μ*g/g) to the dorsal skin of aging mice for 12 months and young control mice for 4 weeks. Electron microscopy analysis of skin samples revealed that LP increased dermal thickness and skin collagen content. Tissue inhibitor of metalloprotease- (TIMP-) 1 expression was upregulated while that of matrix metalloproteinase- (MMP-) 1 was downregulated in skin tissue of LP-treated as compared to untreated aging mice. Additionally, phosphorylation of c-Jun N-terminal kinase (JNK) and p38 was higher in aging skin than in young skin, while LP treatment suppressed phospho-JNK expression. LP application also enhanced the expression of antioxidative enzymes in skin tissue, causing a decrease in malondialdehyde levels and increases in superoxide dismutase, catalase, and glutathione peroxidase levels relative to those in untreated aging mice. These results indicate that LP inhibits MMP-1 expression by preventing oxidative stress and JNK phosphorylation, thereby delaying skin collagen breakdown during aging.

## 1. Introduction

Skin is the largest organ of the human body and serves as a protective barrier from environmental stressors such as heat, infection, water loss, and ultraviolet radiation. In contrast to photoaging, which results from the effects of ultraviolet rays [1], intrinsic aging occurs naturally over time [2]. In addition to environmental factors, genetics, cellular metabolism, hormones, and metabolic processes contribute to natural aging.

The development of age-related skin pathologies is associated with alterations in the levels of collagen in skin extracellular matrix (ECM) [3]. Matrix metalloproteases (MMPs) are the major enzymes involved in ECM degradation. Type I collagen is mainly hydrolyzed by MMPs/collagenases (e.g., MMP-1, MMP-8, and MMP-13). MMP-1 is the predominant collagenase in the skin [4] whose activity is suppressed by tissue inhibitor of metalloproteinase- (TIMP-) 1. Given that the breakdown of collagen is a major cause of wrinkle formation, an obvious manifestation of aging [5, 6], blocking this process by inhibiting MMP activity is a potential strategy for preventing skin aging.

Aging is associated with cellular damage caused by endogenous reactive oxygen species (ROS) [7, 8]. Redox reactions activate c-Jun N-terminal kinase (JNK) signaling, which induces the expression of transcription factors such as activator protein- (AP-) 1 and nuclear factor *κβ* that play important roles in MMP activation [9].

*Laminaria japonica* is a type of brown seaweed that is widely consumed in China. Kelp is used in traditional Chinese medicine [10]; polysaccharides extracted from seaweed have antioxidant [11], anti-inflammatory [12], and antitumorigenic [13] properties. In our previous study, we showed that* Laminaria* polysaccharide (LP) had antioxidative activity in vascular endothelial cells of rats [14]; however, systemic delivery of an antioxidant to the skin is inefficient [15], while topical application can be beneficial if sufficient quantities of the substance penetrate the skin [16, 17]. The present study explored whether topical application of LP can prevent wrinkling of aging skin by blocking collagen degradation.

## 2. Materials and Methods

### 2.1. Chemicals and Reagents

The extract of* Laminaria* polysaccharide (LP) was performed according to our previously reported [14]. Vitamin E (Vit. E) was purchased from Sinopharm Chemical Co. (Shanghai, China). Ointment base was purchased from pharmaceutical factory of Guangxi Medical University (Guangxi, China). It is a washable, oil-in-water emulsion base that contains purified water, petrolatum, cetostearyl alcohol, propylene glycol sodium lauryl sulfate, isopropyl palmitate, imidazolidinyl urea, methylparaben, and propylparaben.

### 2.2. Animals

Specific pathogen-free grade female Kunming mice (18–25 g, 8 weeks old) purchased from the Experimental Animal Center of Guangxi Medical University (Nanning, China) were maintained in a temperature- and humidity-controlled environment on a 12 : 12 h light/dark cycle. Animals were allowed free access to standard laboratory food and water. Animal protocols were approved by the Institutional Animal Care and Use Committee of Guangxi Medical University.

LP was mixed with the ointment base at concentrations of 10, 25, and 50 *μ*g/g. Vit. E was used as a positive control and mixed with ointment base at a concentration of 10 *μ*g/g, since some studies have reported its antioxidant properties and antiaging effects on skin [17, 18]. Mice were divided into six groups: young control and aging models (receiving ointment base without additives); 10, 25, and 50 *μ*g/g LP ointment (LP low (LP-L), middle (LP-M), and high (LP-H) dose, resp.); and 10 *μ*g/g Vit. E. Hair on the back of each mouse was shaved and 0.1 g of ointment was topically applied to a 2 × 2 cm^2^ patch of skin twice daily at 10:00 and 16:00 h for 12 months, except in the young control group (4 weeks). At the end of the experiment, mice were sacrificed by cervical dislocation under isoflurane anesthesia and dorsal skin tissue samples were immediately collected for analysis.

### 2.3. Histological Analysis

Part of each skin sample (1 × 1 cm^2^) was fixed in 4% paraformaldehyde for hematoxylin and eosin staining. The thickness of the dermis was determined with the MIE.3 image processing and analysis system (Echuang Electronics, Shandong, China). Each section was imaged five times and each image was measured five times to obtain an average value.

### 2.4. Biochemical Analysis

Total skin collagen can be determined by evaluating the content of hydroxyproline (HYP), the major constituent amino acid in collagen [19]. HYP levels in the dorsal skin were measured using a HYP detection kit (Nanjing Jiancheng Bioengineering Institute, Nanjing, China) according to the manufacturer's instructions. Malondialdehyde (MDA) [20] level and superoxide dismutase (SOD), catalase (CAT), and glutathione peroxidase (GSH-Px) activity in skin tissue were determined using commercial kits (Nanjing Jiancheng Bioengineering Institute).

### 2.5. Quantitative Real-Time PCR

Total RNA was extracted from dorsal skin tissue of mice (*n*  = 5) using TRIzol reagent (Invitrogen, Carlsbad, CA, USA) as recommended by the manufacturer. Total RNA (2 *μ*g) was reverse transcribed to cDNA using a kit (Takara Bio, Otsu, Japan) according to the manufacturer's protocol. Target genes were amplified by real-time PCR on an ABI Prism 7500 sequence detection system (Applied Biosystems, Foster City, CA, USA) using SYBR Green Real-Time PCR Master Mix (Takara Bio) and the following forward and reverse primer sets: type I collagen (NM_007743.2), 5′-CGA TGT TGA ACT TGT TGC TGA-3′ and 5′-AGG CGA GAT GGC TTA TTT GTT-3′, and *β*-actin (NM_007393. 3), 5′-CAT CCG TAA AGA CCT CTA TGC CAA C-3′ and 5′-ATG GAG CCA CCG ATC CAC A-3′. To confirm the specificity of the amplification, PCR products were evaluated by melting curve analysis. mRNA expression was determined based on cycle threshold values, which were normalized to that of *β*-actin and calculated using the 2^−ΔΔCT^ method [21].

### 2.6. Western Blot Analysis

Skin tissue samples were lysed in radioimmunoprecipitation assay buffer (Beyotime, Shanghai, China) and total protein concentration was measured with a bicinchoninic acid assay kit (Beyotime). Western blotting was performed as previously described [22] using antibodies against the following proteins: MMP-1 (rabbit polyclonal, 1 : 1000, Abcam, Cambridge, UK, cat. number ab137332); TIMP-1 (rabbit polyclonal, 1 : 200, Santa Cruz Biotechnology, Santa Cruz, CA, USA, cat. number 5538); and JNK (rabbit monoclonal, 1 : 1000, cat. number 9252), phospho-JNK (rabbit monoclonal, 1 : 1000, cat. number 4668), p38 mitogen-associated protein kinase (MAPK) (rabbit polyclonal, 1 : 1000, cat. number 9212), and p-p38 (rabbit polyclonal, 1 : 1000, cat. number 9211) (all from Cell Signaling Technology, Danvers, MA, USA). Glyceraldehyde 3-phosphate dehydrogenase (mouse monoclonal, 1 : 20,000, Sigma, St. Louis, MO, USA, cat. number G9295) served as a loading control. Protein band intensity was quantified using Gene Tools image analysis software (Syngene, Cambridge, UK).

### 2.7. Statistical Analysis

Results are expressed as the mean ± SD. Data were analyzed using SPSS 13.0 software (SPSS Inc., Chicago, IL, USA). The significance of differences between groups was evaluated by one-way analysis of variance, and *P* < 0.05 was considered significant.

## 3. Results

### 3.1. LP Treatment Prevents Age-Induced Degradation of Collagen in the Skin

**Figure 1:**
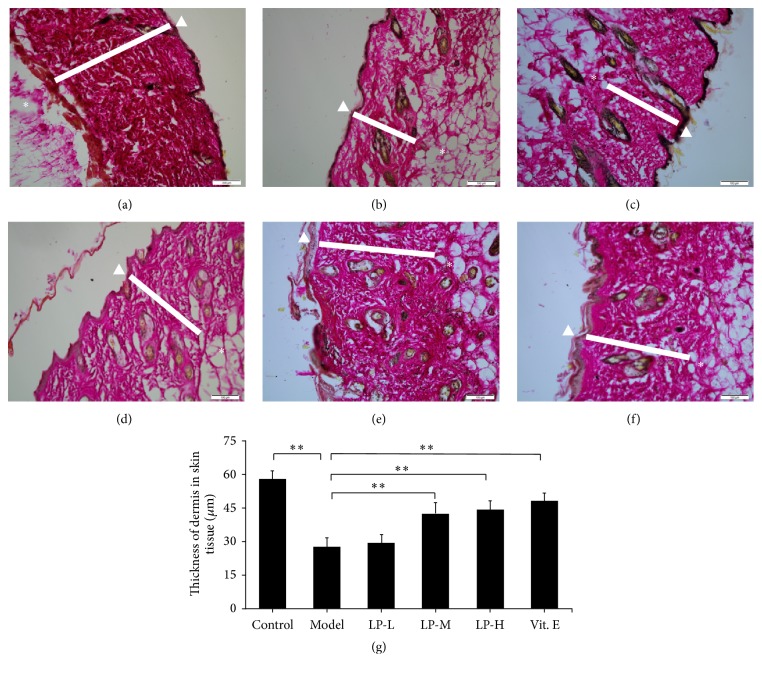
Dermal thickness is restored in aging mice by LP treatment. Hematoxylin and eosin staining of skin tissue samples revealed a decrease in the thickness of the dermis in aging as compared to young mice, which was mitigated by LP or Vit. E treatment. (a) Young control group; (b) aging model; (c) LP-L; (d) LP-M; (e) LP-H; and (f) Vit. E. Bar: 200 *μ*m. The epidermis is indicated with a white triangle, the dermis with a white rectangle, and the hypodermis with an asterisk. (g) Quantitative analysis of dermal thickness (*n* = 5). Values represent mean ± SD. ^*∗∗*^ *P* < 0.01 versus aging model group.

**Figure 2:**
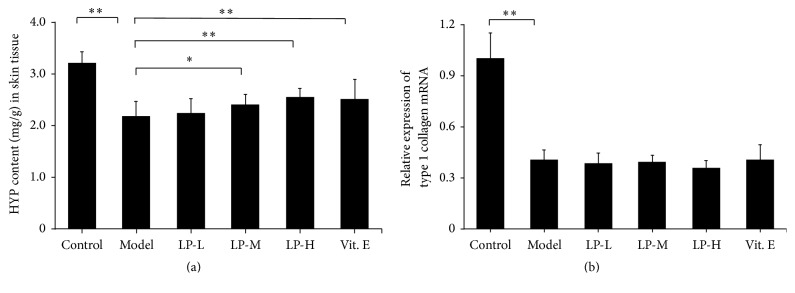
LP increases collagen content in the skin of aging mice. (a) HYP content (*n* = 12) and (b)* type I collagen* mRNA levels (*n* = 5) were compared between young control, aging model, LP-L, LP-M, LP-H, and Vit. E groups. Values represent mean ± SD. ^*∗*^ *P* < 0.05 and ^*∗∗*^ *P* < 0.01 versus aging model group.

To investigate the effect of LP on collagen in aging skin, we assessed the thickness of the dermis (Figure 1) and HYP content of skin tissue which were reduced in aging as compared to young mice; however, LP treatment increased both dermal thickness and skin HYP content in a dose-dependent manner relative to aging mice without treatment (Figure 2(a)). In addition,* type I collagen* mRNA expression was reduced in the aging model relative to young mice but did not differ between aging mice with or without LP or Vit. E treatment (Figure 2(b)).

### 3.2. LP Treatment Modulates MMP-1 and TIMP-1 Expression in the Skin of Aging Mice

**Figure 3:**
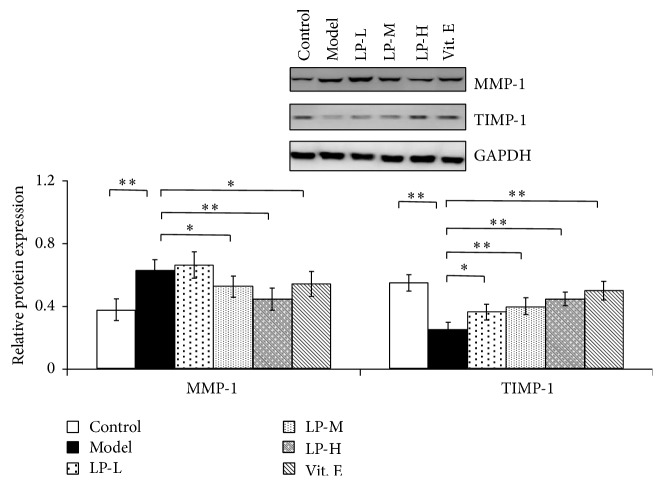
LP treatment modulates MMP-1 and TIMP-1 levels in aging skin tissue. MMP-1 and TIMP-1 levels in skin tissue of young mice or aging mice without or with LP-L, LP-M, LP-H, or Vit. E treatment, as determined by western blotting. Glyceraldehyde 3-phosphate dehydrogenase (GAPDH) served as a loading control. Expression levels were quantified by densitometry. Values represent mean ± SD (*n* = 5). ^*∗*^ *P* < 0.05 and ^*∗∗*^ *P* < 0.01 versus aging model group.

Skin collagen degradation is mainly regulated by MMP-1, which is inhibited by TIMP-1. MMP-1 protein expression was increased in aging as compared to young skin tissue (Figure 3) but was decreased in LP-M, LP-H, and Vit. E groups relative to untreated aging mice. Conversely, TIMP-1 protein level was decreased in aging as compared to young skin tissue, whereas LP treatment caused a dose-dependent increase in TIMP-1 expression relative to untreated aging mice.

### 3.3. LP Inhibits the Age-Induced Increase in JNK and p38 MAPK Signaling

**Figure 4:**
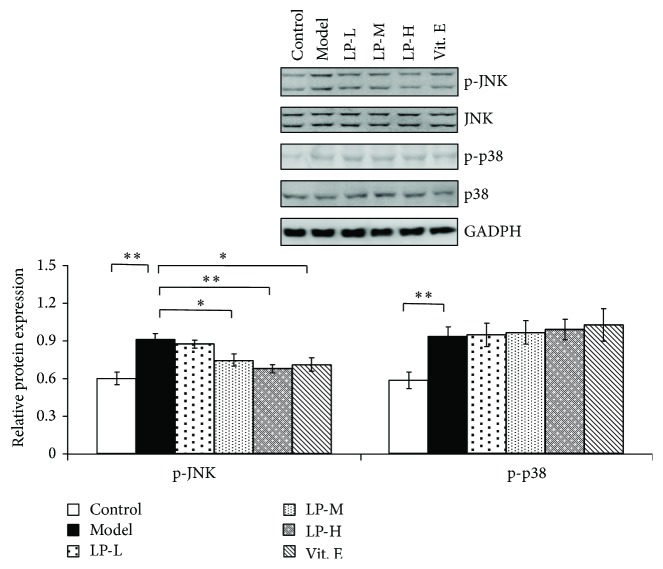
LP reverses the age-induced increase in JNK but not p38 MAPK signaling. Expression of p-JNK, JNK, p-p38, and p38 proteins in skin tissue of young control mice and aging mice without or with LP-L, LP-M, LP-H, and Vit. E treatment, as determined by western blotting. Glyceraldehyde 3-phosphate dehydrogenase (GAPDH) served as a loading control. Expression levels were quantified by densitometry. Values represent mean ± SD (*n* = 5). ^*∗*^ *P* < 0.05 and ^*∗∗*^ *P* < 0.01 versus aging model group.

To investigate the molecular mechanisms underlying the skin aging process, we examined the activation of JNK and p38 MAPK signaling pathways in aging skin with or without LP treatment by western blotting. The level of p-JNK increased with aging; however, this was abrogated by application of LP or Vit. E (Figure 4). Similarly, p-p38 level was upregulated in aging as compared to young mice; however, LP or Vit. E application did not alter the level relative to untreated mice.

### 3.4. LP Treatment Stimulates Antioxidant Enzyme Expression in Aging Skin

**Figure 5:**
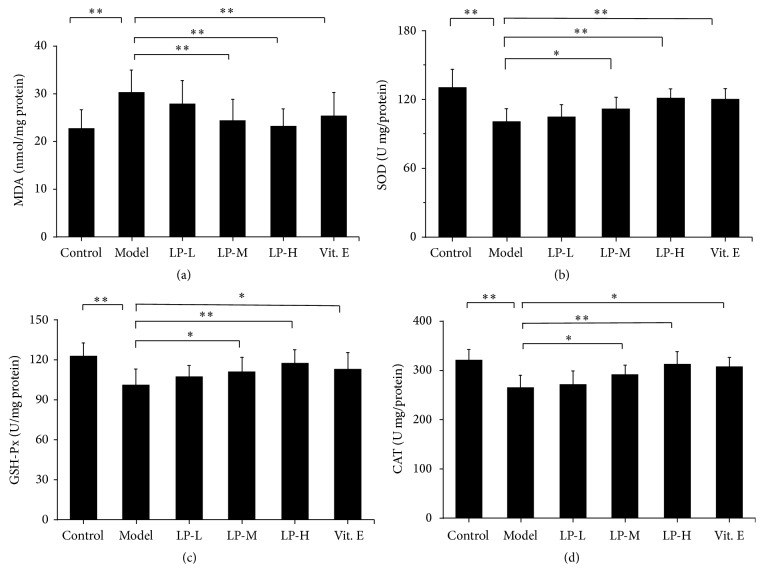
Antioxidant enzyme expression is upregulated by LP treatment in aging skin. Expression levels of (a) MDA, (b) SOD, (c) GSH-Px, and (d) CAT in the skin tissue of young control mice and aging mice without or with LP-L, LP-M, LP-H, or Vit. E treatment, as determined by western blotting. Values represent mean ± SD (*n* = 12). ^*∗*^ *P* < 0.05 and ^*∗∗*^ *P* < 0.01 versus aging group.

Since JNK phosphorylation can be stimulated by ROS, we investigated the expression of antioxidative enzymes in aging skin with or without LP treatment. Compared to young mice, MDA level was increased in the dorsal skin of aging mice; however, this was attenuated in the LP-M, LP-H, and Vit. E groups relative to the aging model group (Figure 5(a)). Conversely, SOD, GSH-Px, and CAT levels were downregulated in aging relative to young mice, and LP or Vit. E application could reverse the level relative to untreated mice (Figures 5(b)–5(d)).

## 4. Discussion

We demonstrated in this study that topical application of LP can alleviate the alterations in collagen in the skin that are induced by aging and regulate the balance between MMP-1 and TIMP-1 by inhibiting JNK phosphorylation. Moreover, we found that LP treatment increased the levels of antioxidant enzymes in the skin, which likely suppresses the levels of ROS that also contribute to the breakdown of collagen, leading to wrinkling.

During the aging process, the dermis of the skin becomes thin and damaged [23] due to the degradation of the collagen matrix [24, 25]. The amount of fragmented collagen is about 4-fold greater in the dermis of individuals >80 years old as compared to those who are 21–30 years old [26]. Type I collagen is the most abundant protein in human skin, comprising about 90% of the dry weight, but the level decreases gradually over the course of a lifetime [26], resulting in a 20%–80% decrease in the thickness of the dermis. In this study, topical application of LP did not stimulate type I collagen production in aging skin but prevented decreasing of the thickness of the dermis.

MMP-1 expression has been shown to increase with age [27, 28], which is a major factor in the breakdown of collagen and skin wrinkling [6]. In this study, LP treatment suppressed the aging-induced upregulation of MMP-1 protein expression. It has been reported that plasma TIMP-1 level is decreased in aged individuals [29]. We confirmed downregulation in TIMP-1 expression in the skin of aging mice and found that LP treatment abrogated this effect as compared to aged mice skin without treatment. These results suggest that LP maintains the balance between MMP-1 and TIMP-1, which is important for the maintenance of ECM homeostasis.

MAPK family proteins include JNK, p38, and extracellular signal-regulated kinase (ERK). Age-associated MMP-1 expression is ROS dependent and regulated by activation of JNK signaling [30]. JNK is a serine threonine kinase that phosphorylates c-Jun, a component of the AP-1 complex [31]. c-Jun levels were increased in human dermal fibroblasts from individuals >80 years old relative to young individuals [32]. We found that LP treatment inhibited JNK phosphorylation, which likely led to the suppression of MMP-1 in aging skin. Application of a p38 inhibitor reportedly increased rubratoxin B-induced TIMP-1 secretion, which was not blocked in the presence of JNK inhibitor [33]. In the present study, p38 activation was increased in aging skin, which was accompanied by downregulation in TIMP-1 protein expression; LP treatment countered this decrease but did not inhibit p38 phosphorylation, indicating that it regulated TIMP-1 expression through a different signaling pathway.

Aging is primarily a consequence of aerobic metabolism, which produces ROS in excess of cellular antioxidant defense capacity [34]. Oxidants are important mediators of aging [35, 36]; indeed, ROS production is linked to the age-associated increase in MMP-1 levels [27, 32]. Oxidative conditions in the cell generate ROS that induce MMP-1 synthesis via activation of JNK/AP-1 signaling [37]. MDA is a marker for damage to cell membrane phospholipids caused by free radicals [38]. Antioxidant enzymes in the skin including CAT, SOD, and GSH-Px counter ROS [39]; treatment of primary dermal fibroblasts from photoaged skin with CAT reversed aging-induced MAPK changes and inhibited MMP-1 expression via activation of JNK signaling [40]. In our study, LP not only decreased the expression of MDA but also increased those of SOD, CAT, and GSH-Px. Hence, it is possible that LP improves the antioxidative capacity of aging skin by suppressing JNK phosphorylation and consequently inhibiting MMP-1 activity.

## 5. Conclusion

In summary, our findings indicate that topical application of LP can enhance the antioxidative capacity of aging skin in a mouse model. This effect resulted in the suppression of JNK signaling phosphorylation/activation and the consequent restoration of the balance between MMP-1 and TIMP-1 levels, which could delay the breakdown of collagen. These findings provide a basis for the use of LP in antiaging agent products for the skin, while additional studies are needed to confirm the effect of LP on antiaging signaling pathways.
